# Cross‐mapping medical records to NANDA‐I to identify nursing diagnoses in a vulnerable population

**DOI:** 10.1111/2047-3095.12371

**Published:** 2022-04-22

**Authors:** Domingo Ángel Fernández‐Gutiérrez, Pedro Ruymán Brito‐Brito, Sara Darias‐Curvo, Antonio Cabrera‐de‐León, Carlos Enrique Martínez‐Alberto, Armando Aguirre‐Jaime

**Affiliations:** ^1^ Associate Professor in the Nursing Department Universidad de La Laguna and Health Care Information Systems Facilitator and Blended‐Learning Advisor in Primary Health Care Management Servicio Canario de la Salud. Member of the University Study Center for Social Inequalities and Governance, CEDESOG Universidad de La Laguna, Tenerife Canary Islands Spain; ^2^ Associate Professor in the Nursing Department Universidad de La Laguna, and Research Nurse in Primary Care Management Servicio Canario de la Salud, Tenerife Canary Islands Spain; ^3^ Full Professor in the Nursing Department Universidad de La Laguna, Tenerife. Member of the University Study Center for Social Inequalities and Governance, CEDESOG. Canary Islands Spain; ^4^ Research Physician in the Research Unit of Ntra. Sra. de Candelaria Hospital Tenerife. Full professor of Preventive Medicine and Public Health Universidad de La Laguna Canary Islands Spain; ^5^ Associate Professor in the Nursing Department European University of the Canary Islands Laureate International Universities and Research Nurse in Primary Care Management Servicio Canario de la Salud, Tenerife Canary Islands Spain; ^6^ Research Advisor Care Research Institute Colegio de enfermeros de Santa Cruz de Tenerife Department of Public Health European University of the Canary Islands Laureate International Universities, Tenerife Canary Islands Spain

**Keywords:** Cross‐mapping, nursing care, nursing epidemiology, social determinants of health, standardized nursing terminology

## Abstract

**Purpose:**

To assess the association between vulnerable populations and nursing care needs, using NANDA‐I diagnostics, in the population of the Canary Islands, Spain.

**Methods:**

Nursing social epidemiology study. Cross Mapping of Medical Records to NANDA‐I to Identify Nursing Diagnoses in a Population usinga medical, epidemiological follow‐up study of a cohort of 7,190 people. The level of vulnerability of the participants was assigned, among those who were also assigned nursing diagnoses, using the “ICE index” to calculate the expected associations.

**Findings:**

The most prevalent nursing diagnosis in our sample was Sedentary lifestyle (60.5%), followed by Ineffective health self‐management (33.8%) and Risk‐prone health behaviour (28.7%). Significant differences were found by sex, age group and social class, with the nursing diagnoses included in the study being more prevalent among the most socio‐economically disadvantaged social class.

**Conclusions:**

: The cross‐mapping method is useful to generate diagnostic information in terms of care needs, using the NANDA‐I classification. The expected associations between high social vulnerability and care needs have been verified in a comprehensive and representative sample of the Canarian population (Spain).

**Implications for nursing practice:**

From an epidemiological perspective, identifying nursing diagnoses at the population level allows us to find the most prevalent needs in the different community groups and to focus appropriate nursing interventions for their implementation and impact assessment.

## Resumen

**Objetivo:**

Evaluar la asociación entre las poblaciones vulnerables y las necesidades de cuidados de enfermería, utilizando la clasificación diagnóstica NANDA‐I, en la población de las Islas Canarias, España.

**Métodos:**

Estudio de epidemiología social enfermera. Mapeo cruzado de registros médicos con la clasificación NANDA‐I para identificar los diagnósticos de enfermería en una población mediante un estudio de seguimiento médico y epidemiológico de una cohorte de 7.190 personas. Se asignó el nivel de vulnerabilidad de los participantes, entre los que también se asignaron diagnósticos de enfermería, utilizando el “índice REI” para calcular las asociaciones esperadas.

**Resultados:**

El diagnóstico de enfermería más prevalente en nuestra muestra fue Estilo de vida sedentario (60,5%), seguido de Autogestión ineficaz de la salud (33,8%) y Tendencia a adoptar conductas de riesgo para la salud (28,7%). Se encontraron diferencias significativas por sexo, grupo de edad y clase social, siendo los diagnósticos de enfermería incluidos en el estudio más prevalentes entre la clase social más desfavorecida socioeconómicamente.

**Conclusiones:**

El método de mapeo cruzado es útil para generar información diagnóstica en términos de necesidades de cuidados, utilizando la clasificación NANDA‐I. Se han verificado las asociaciones esperadas entre alta vulnerabilidad social y necesidades de cuidados en una muestra amplia y representativa de la población canaria (España).

**Implicaciones para la práctica enfermera:**

Desde una perspectiva epidemiológica, la identificación de los diagnósticos de enfermería a nivel poblacional permite encontrar las necesidades más prevalentes en los diferentes grupos de la comunidad y focalizar las intervenciones enfermeras adecuadas para su implementación y evaluación de impacto.

## PURPOSE

Care needs are the central object of nursing diagnoses that require critical reasoning in order to make a clinical judgment about human responses to health and illness. Nursing diagnosis allows nurses to organize care and focus on identifying and prioritizing care needs of an individual or population. The most prevalent classification of nursing diagnoses worldwide is NANDA‐I (Herdman et al., 2021). This diagnostic language has great importance in identifying and labeling not only an individual's nursing care, but also priority nursing care needs for different population groups (Brito‐Brito et al., 2016).

The starting hypothesis of this research work is that nursing care needs, identified according to the NANDA‐I classification, are more prevalent among people who experience socioeconomic vulnerabilities. In line with this approach, this study aims to assess the association between such vulnerable populations in the Canary Islands of Spain and nursing care needs, using NANDA‐I diagnoses.

## BACKGROUND

Primary health care (PHC) nurses in the public health service of the Canary Islands, Servicio Canario de la Salud (SCS), use the NANDA‐I classification of nursing diagnoses to record, in electronic health records, the care needs they identify in the community context.

According to the Spanish law, Ley 14/1986, General de Sanidad, and the Spanish Constitution, article 43, all the residents in Spain, no matter if legal or not, have the right to public healthcare assistance. The population assigned to the SCS is around 2,000,000 people. However, 36% were at risk of economic vulnerability or social exclusion in 2018 (National Institute of Statistics, 2019). This level is 10% higher than the national average and the third‐highest rate out of the 17 health regions in Spain.

In the 19th century, Virchow illustrated the association of a higher frequency of illness and death as a consequence of people's economic positions (Bohenheim, 1957). Vulnerability from a health perspective includes limited access to economic resources, marginalized sociocultural status, and personal characteristics such as age, gender, occupation, education, and environmental accessibility to health services (Cohen et al., 2017; De Chesnay & Anderson, 2019). Promoting health resources makes it possible to modulate social differences by counteracting them to enhance individual and community capabilities in disadvantaged environments (Roy et al., 2018).

Current efforts to identify and address social inequalities in health have produced guidance documents such as the Lalonde Report (Lalonde, 1974) and the Report of the Working Group on Inequalities in Health (Townsend et al., 1988). Morbidity and mortality are higher among vulnerable populations, and it is known that there is a gradient in health that is inversely proportional to the level of vulnerability and social position (Marmot, 2017). Implicit in the concept of social determinants of health is the fact that disease and health are not randomly distributed among social groups nor are the resources allocated to disease prevention (Havranek et al., 2015).

Some studies have identified the most prevalent nursing diagnoses in socioeconomically disadvantaged populations (Da Silva et al., 2018) and, more recently, a nursing diagnosis has been proposed for people experiencing unemployment (Bocchino et al., 2017). However, the relationships between the level of vulnerability and the prevalence of nursing care needs have not been described in large and representative population samples using the NANDA‐I diagnostic terminology.

## METHOD

This is a nursing, social epidemiology study (Brito‐Brito et al., 2016) applying the cross‐mapping method for the identification of NANDA‐I diagnoses on data from the “CDC‐Canarias” cohort (Cabrera de León et al., 2008). “CDC‐Canarias” is a medical, epidemiological follow‐up study of a cohort of 7,190 people, representative of the age, sex, and geographical distribution of the Canary Islands population to identify risk factors for cancer, diabetes, and cardiovascular diseases.

### Data collection procedure

Cross‐mapping allows comparing terms from different terminologies to determine their semantic equivalence (D´Agostino et al., 2020; Goossen, 2006).

The “CDC‐Canarias” database has 166 information items distributed in 956 fields for the period 2000–2016. The procedure for using these data consisted of five phases:

1. The exploration and identification of data for formulating NANDA‐I nursing diagnoses were performed. A priori 170 variables were selected to be combined by cross‐mapping.
2. A cross‐mapping was performed with variables and combinations with NANDA‐I labels, their defining characteristics, related factors, risk factors, at risk population, and associated conditions. Semantic equivalences (Frauenfelder et al., 2016) were validated by the degree of consensus in a focus group of eight experts. To consider members of the focus group as experts, their training and care experience were taken into account, and they had to meet at least one of the following four criteria: to have a minimum of 50 hours of formal training in nursing methodology, to have at least five years' experience of clinical practice, and teaching or research with standardized nursing languages (Lopes et al., 2010). The consensus of the experts was explored in successive rounds until at least a 75% agreement level was reached.
3. NANDA‐I diagnoses obtained are attributed to the “CDC‐Canarias” sample subjects. The consistency of these assignments is verified in each subsample by reproducing known relationships of these diagnoses with health problems and demographic characteristics.
4. The income, crowding index, and education (ICE) index is a validated quantitative indicator not based on occupation or employment status to classify the adult population according to their social vulnerability and its impact on health (Cabrera de León et al., 2009). The ICE index is assigned to the subjects of the “CDC‐Canarias” sample, selecting those belonging to the most vulnerable range and the less vulnerable ones for the present study. In this subsample, the verification of the assignment's consistency is achieved by replicating known associations between vulnerability, age, sex, and suffering from chronic diseases.
5. The determination of associations between NANDA‐I diagnoses and social vulnerability in each subsample is established with the diagnoses mentioned in point 3 and with the ICE index in point 4. Prechecks on the consistency of NANDA‐I label assignments and ICE scores support the credibility of these associations.

### Variables and instruments

Variables contained in the “CDC‐Canarias” database used were:

- Sociodemographics: sex, age, education, family income per capita.
- Lifestyle: smoking, alcohol consumption, daily physical activity during work and leisure time, Hours of nightly sleep, excessive perceived stress.
- Family medical history: health problems suffered by father, mother, paternal or maternal grandparents.
- Diet: adherence to the Mediterranean diet, eating out or eating in between meals daily, consumption of sugary drinks per week, fluid intake below daily recommendations.
- Health problems and adherence to treatment: diagnosed diseases, risk factors for cancer, diabetes or cardiovascular problems, adherence to prescribed treatments.
- Level of vulnerability: The score on the ICE index (Cabrera de León et al., 2009) assigned to the subjects in the sample (4–21 points) is stratified into quintiles, selecting the two lowest extremes as a most vulnerable and the highest as less vulnerable, in accordance with the distribution of the Canarian population at risk of poverty and social exclusion and with a medium‐high socioeconomic level (National Institute of Statistics, 2019).

### Data analysis

Data were analyzed with IBM SPSS v.24.0. The characteristics of the sample were described by summarizing nominal variables with the absolute and relative frequencies of their categories. The scale variables are expressed with the mean (SD), as they follow a normal distribution verified by the Kolmogorov–Smirnov test. The consistency of the NANDA‐I labels and the ICE index assignment was verified by checking the replication of their known correlates with other characteristics of the subjects by means of the Pearson χ^2^ test or the Fisher exact test when necessary. The associations between ICE index social vulnerability and NANDA‐I diagnoses were estimated in the same way. These associations were corroborated by estimating the crude and age‐ and sex‐adjusted relative risks of receiving a NANDA‐I diagnostic label for having the condition of vulnerability using binary logistic regression models with sex and age as covariates. Statistical significance was set at *P* < 0.05.

### Ethical considerations

The “CDC‐Canarias” study was approved by the Ethics Committee of the NS Candelaria University Hospital (Tenerife, Canary Islands, Spain). All participants gave their written informed consent where they were asked for authorization to use both the data collected through questionnaires and microbiological laboratory analytical tests and biological samples preserved anonymously in other studies nested in the follow‐up of the cohort.

## RESULTS

Fifty‐six percent of the 7,190 people in the initial “CDC‐Canarias” sample were women, and the average age was 43, with 13% being 60 or older. Forty‐one percent of the subjects suffered from respiratory diseases, 28% from obesity, 20% from high blood pressure (HBP), 16% from diabetes mellitus (DM), 2% from cancer, 2% from acute myocardial infarction (AMI), and 1% from cerebral thrombosis or hemorrhage. Thirty percent had other health problems, and 41% had been in hospital for more than 24 hours in the previous year. The remaining sociodemographic and clinical characteristics, exposure factors, and other issues of medical–epidemiological interest of the “CDC‐Canarias” sample have been previously published (Cabrera de León et al., 2008) and are not used here as they are not the subject of this study.

The ICE index score for the subjects in the sample assigned 2,876 of them to the quintiles corresponding to the most vulnerable and 1,438 to the quintile of less vulnerable. The verification of the consistency of the level of vulnerability assignment to the subjects of this subsample with respect to health problems produced, for HBP, 748 cases (26%) among the most vulnerable compared with 187 (13%) among the less vulnerable (*P* < 0. 001); for DM, 316 cases (11%) versus 43 (3%) (*P* < 0.001); for AMI, 115 cases (4%) versus 29 (2%) (*P* < 0.001); and for obesity, 1,007 cases (35%) versus 201 (14%) (*P* < 0.001). The age, by group, of the 4,314 subjects belonging to the quintiles to be compared according to level of vulnerability was distributed for the most vulnerable in 903 (21%) in the 18–39‐year‐old age group, 1,556 (36%) in the 40–59‐year‐old age group and 417 (90%) in the 60‐year‐old or more age group (*P* < 0.001), with the correlation between age as a continuous variable and ICE index for this subsample of −0.28 (*P* < 0.001). There was not, as expected, a higher prevalence of vulnerability in women.

With the information available in the database and after applying cross‐mapping, 15 diagnostic statements were obtained from the 2021–2023 NANDA‐I classification. The nursing diagnoses were constructed by the final combination of 31 main variables (Table 1). A total of eight problem‐focused diagnoses were formulated, one of which was community‐based, and seven risk diagnoses. Table 1 shows the identified diagnoses, the component aspects of these diagnoses contained in the database, the corresponding diagnostic indicators in NANDA‐I, the rules for obtaining these diagnoses, the number of rounds held to arrive at a consensus on all these aspects, and the degree of agreement achieved in the last round.

**Table 1:** Correspondence of diagnostic rules between the component aspects included in the “CDC‐Canarias” study survey and NANDA‐I diagnostic indicators

	NANDA‐I diagnostic label	Component aspects contained in the database “CDC‐Canarias”	Diagnostic indicators of correspondence in NANDA‐I	Diagnostic rule	Rounds held and degree of consensus (%)
Domain 1	00276 Ineffective health self‐management	‐ Inadequately taking (seasonal or daily yes/no) treatments for HBP, DM, or hypercholesterolemia. ‐ Poor blood pressure control at the time of the examination.	‐ Failure to include treatment regimen in daily living (DC). ‐ Exacerbation of disease signs (DC).	Presence of at least one of the two aspects considered.	3 (88%)
1	00292 Ineffective health maintenance behaviors	‐ Not eating well (no adherence to Mediterranean diet) ‐ Not sleeping well (<6 hours/day). ‐ Recommended physical activity (moderate or vigorous or intensive ≥3 METs, equivalent to brisk walking). ‐ Smoking. ‐ Excessive alcohol consumption. ‐ Nonadherence to prescribed treatments.	‐ Failure to take action that prevents health problem (DC). ‐ Failure to take action that reduces risk factor (DC). ‐ Ineffective choices in daily living for meeting health goal (DC). ‐ Pattern of a lack of health‐seeking behavior (DC).	Presence of at least one of the two aspects considered.	5 (75%)
1	00168 Sedentary lifestyle	‐ Actively consume less than 10% of total energy expenditure by not taking at least 25–30 minutes of active leisure time daily. Declared physical activity, anthropometry, and biochemical markers of cardiovascular risk are included.	‐ Average daily physical activity is less than recommended for age and gender (DC).	Presence according to “CDC‐Canarias” criteria.	1 (100%)
1	00188 Risk‐prone health behavior	‐ Smoking and excessive alcohol consumption (>50 g/day in men and >25 g/day in women)	‐ Failure to take action that prevents health problem (DC).	Presence of at least one of the two aspects considered.	3 (88%)
1	00215 Deficient community health	‐ Have DM. ‐ Have HBP. ‐ Cancer disease. ‐ A respiratory disease. ‐ Bleeding‐thrombosis disease. ‐ Angina‐infarction disease	‐ Health problem experienced by groups or populations (DC). ‐ Risk of physiological manifestations to a group or population (DC). ‐ Risk of hospitalization to a group or population (DC).	Presence of at least one of the two aspects considered.	6 (75%)
1	00231 Risk for frail elderly syndrome	‐ Age ≥65 years. ‐ Anorexia (BMI ≤18.5 kg/m^2^). ‐ Endocrine regulation dysfunction, in the presence of diagnosed DM or high blood glucose. ‐ Individual monthly income lower than <850 euros. ‐ DM, HBP, cancer, respiratory, or cardiovascular problems (hemorrhages or heart attacks). ‐ Overcrowding index (person‐per‐bedroom) to consider reduced living space (≥3). ‐ Physical activity of 30 minutes per day at least five days a week. ‐ Low educational level, illiteracy, or primary schooling ‐ Obesity (BMI >30 kg/m^2^). ‐ Being a woman. ‐ Living alone.	‐ Individuals aged > 70 years (modified for the study population to ≥65 years according to the senior's health care program of the Canary healthcare system) (ARP). ‐ Anorexia (AC). ‐ Endocrine regulatory dysfunction (AC). ‐ Economically disadvantaged individuals (ARP). ‐ Chronic disease (AC). ‐ Individuals living in constricted spaces (ARP). ‐ Sedentary lifestyle (RF). ‐ Malnutrition (RF). ‐ Individuals with low educational level (ARP). ‐ Socially vulnerable individuals (ARP). ‐ Obesity (RF). ‐ Women (ARP). ‐ Individuals living alone (ARP).	Presence of at least four of the 10 aspects considered, with the person being 65 years of age or older.	4 (88%)
2	00179 Risk for unstable blood glucose level	‐ DM disease. ‐ Cancer, heart attack, or stroke. ‐ Overwhelmed by lack of time or rushing. ‐ Organ involvement due to DM: kidney, heart, vision, lower limbs, circulation. ‐ Not taking treatment for diabetes regularly (every other day; seasonal). ‐ Physical activity of 30 minutes a day at least five days a week.	‐ Inadequate adherence to treatment regimen (RF). ‐ Individuals with compromised physical health status (ARP). ‐ Excessive stress (RF). ‐ Inadequate diabetes self‐management (RF). ‐ Ineffective medication self‐management (RF). ‐ Sedentary lifestyle (RF).	Presence of DM and at least two of the other five aspects considered.	2 (100%)
2	00232 Obesity	‐ IMC >30 kg/m^2^.	‐ ADULT: Body mass index > 30 kg/m^2^ (DC).	Presence of the aspect under consideration.	1 (100%)
2	00233 Overweight	‐ IMC 25–30 kg/m^2^.	‐ ADULT: Body mass index > 25 kg/m^2^ (DC).	Presence of the aspect under consideration.	1 (100%)
2	00234 Risk for overweight	‐ 23 kg/m^2^ ≤ BMI ≤−25 kg/m^2^. ‐ Lunch or dinner in bars or restaurants daily. ‐ Eating between meals. ‐ Consuming soft drinks more than two times a week (still/fizzy). ‐ Presence of sedentariness according to “CDC‐Canarias” criteria. ‐ Excessive alcohol consumption. ‐ Maternal DM. ‐ Sleep problems (<6 hours of nightly rest).	‐ ADULT: Body mass index approaching 25 kg/m^2^ (ARP). ‐ High frequency of restaurant or fried food (RF). ‐ Frequent snacking (RF). ‐ Consumption of sugar‐sweetened beverages (RF). ‐ Sedentary behavior occurring for ≥ 2 hours/day (RF). ‐ Average daily physical activity is less than recommended for age and gender (RF). ‐ Excessive alcohol consumption (RF). ‐ Individuals whose mothers have diabetes (ARP). ‐ Shortened sleep time (RF).	Presence of at least two of the eight aspects considered.	5 (75%)
3	00011 Constipation	‐ Subject's description about constipation.	‐ Evidence of symptoms in a standardized diagnostic criteria (DC). ‐ Altered regular routine (RF).	Presence of the aspect under consideration.	1 (100%)
3	00015 Risk for constipation	‐ Liquid intake lower than recommended (out of total liquids ingested daily, in cubic centimeters) ‐ Physical activity of 30 minutes a day at least five days a week. ‐ Criteria of sedentarism. ‐ Obesity.	‐ Insufficient fluid intake (RF). ‐ Average daily physical activity is less than recommended for ager and gender (RF). ‐ Endocrine system diseases (AC).	Presence of at least two of the four aspects considered.	5 (75%)
4	00299 Risk for decreased activity intolerance	‐ Suffering from a respiratory disease. ‐ Loss of physical condition defined as obesity, sedentarism. ‐ History of angina, heart attack, or cerebral thrombosis.	‐ Respiration disorders (AC). ‐ Physical deconditioning (RF). ‐ Sedentary lifestyle (RF). ‐ Impaired physical mobility (RF).	Presence of at least two of the three aspects considered.	4 (75%)
4	00311 Risk for impaired cardiovascular function	‐ History of angina, heart attack, or cerebral thrombosis ‐ Suffering from cerebral thrombosis, infarct, angina by father, mother, maternal grandparents, or paternal grandparents. ‐ DM. ‐ Dyslipidemia (cholesterol >200 mg/dl and/or triglycerides >150 mg/dl). ‐ Age > 65 years. ‐ HBP (BP >139/89). ‐ Obesity (BMI >30 kg/m^2^). ‐ Sedentary lifestyle (according to “CDC‐Canarias” criteria). ‐ Smoking by declaration of the subject.	‐ Individuals with history of cardiovascular event (ARP). ‐ Diabetes mellitus (AC). ‐ Dyslipidemia (AC). ‐ Older adults (ARP). ‐ Hypertension (AC). ‐ Body mass index above normal range for age and gender (RF). ‐ Average daily physical activity is less than recommended for ager and gender (RF). ‐ Smoking (RF).	Presence of at least three of the eight aspects considered.	7 75%)
11	00303 Risk for adult falls	‐ Age ≥ 65 years. ‐ Living alone. ‐ Having cancer (including leukemia). ‐ Excessive alcohol consumption ‐ DM.	‐ Individuals aged ≥ 60 years (ARP). ‐ Individuals living alone (ARP). ‐ Individuals in palliative care settings (ARP). ‐ Substance misuse (RF). ‐ Endocrine system diseases (AC).	Presence of at least two of the five aspects considered.	6 (88%)

Abbreviations: HBP, high blood pressure; DM, diabetes mellitus; MET, metabolic equivalent of task; BMI, body mass index; CVA, cerebrovascular accident; DC, defining characteristics; RF, risk factors; ARP, at risk population; AC, associated condition.

The number of subjects to whom the NANDA‐I diagnostic labels derived from the previous phase could be assigned varied depending on the target population of the diagnosis and the availability of the necessary data on the subjects in the “CDC‐Canarias” database. These assignments ranged from a minimum of 184 (2.6% of the sample) for the risk of frail elderly syndrome (00231) label to a maximum of 4,352 (60.5%) for Sedentary lifestyle (00168). Details of the number of assignments that could be made for each of the 15 diagnoses are shown in the first data column of Table 2, which presents the results of the consistency check of the assignment of these labels with respect to sex and age.

**Table 2:** Results of assignment consistency tests for NANDA‐I diagnostics, to the sample subjects by checking for replication of their known associations with sex and age

Code and Diagnostic label	Total*n* (%)	Women*n* (%)	Men*n* (%)	*P* ^1^	Age 18–39*n* (%)	Age 40–64*n* (%)	Age ≥65*n* (%)	*P* ^1^
00276 Ineffective health self‐management	1748 (24.5)	812 (20.2)	936 (30.0)	<0.001	278 (9.0)	1971 (35.5)	99 (41.1)	<0.001

00292 Ineffective health maintenance behaviors	2412 (33.8)	1193 (29.7)	1219 (39.9)	<0.001	1038 (33.5)	1302 (33.7)	72 (29.9)	<0.001
00168 Sedentary lifestyle	4352 (60.5)	2706 (67.4)	1646 (57.2)	<0.001	1680 (54.3)	2527 (65.5)	145 (60.2)	<0.001

00188 Risk‐prone health behavior	2067 (28.7)	865 (21.5)	1202 (38.5)	<0.001	976 (31.5)	1057 (27.4)	34 (14.1)	<0.001
00215 Deficient community health	1140 (16.0)	677 (16.9)	463 (14.8)	0.020	133 (4.3)	931 (24.1)	76 (31.5)	<0.001

00231 Risk for frail elderly syndrome^2^	184 (2.6)	116 (2.9)	68 (2.2)	0.060	‐	‐	184 (76.3)	<0.001
00179 Risk for unstable blood glucose level	270 (3.8)	160 (4.0)	110 (3.5)	0.309	31 (1.0)	218 (5.7)	21 (8.7)	<0.001

00232 Obesity	1966 (27.5)	1138 (28.3)	828 (26.5)	0.086	515 (16.6)	1399 (36.3)	59 (24.5)	<0.001
00233 Overweight	2658 (37.2)	1295 (32.2)	1363 (43.6)	<0.001	987 (31.9)	1596 (41.4)	87 (36.1)	<0.001

00234 Risk for overweight	843 (11.8)	459 (11.4)	384 (11.8)	0.262	581 (18.8)	251 (6.5)	11 (4.6)	<0.001
00011 Constipation	1820 (25.5)	1424 (35.5)	396 (12.7)	<0.001	739 (29.9)	1040 (27.0)	41 (17.0)	<0.001

00015 Risk for constipation	340 (4.8)	165 (4.1)	175 (5.6)	0.003	45 (1.5)	258 (6.7)	37 (15.4)	<0.001
00299 Risk for decreased activity intolerance	1404 (19.7)	832 (20.7)	572 (18.3)	0.011	604 (19.5)	762 (19.8)	46 (19.1)	0.947

00311 Risk for impaired cardiovascular function^3^	1226 (17.2)	693 (17.3)	533 (17.1)	0.829	157 (5.1)	995 (25.1)	74 (30.7)	<0.001
00303 Risk for adult falls	1093 (15.3)	594 (14.8)	499 (16.0)	0.169	366 (11.8)	693 (18.0)	39 (16.2)	<0.001

^1^ Estimated with Pearson chi‐squared test.

^2^ This label only includes persons 60 years of age or older. Age included in the components.

^3^ Age included in some component of the diagnosis, which affects its significance

Of the 4,314 subjects that made up the “CDC” subsample for the most vulnerable and less vulnerable, the subjects with an assignment of the diagnostic labels derived from this base ranged from 132 (1.8% of the total sample) for risk for frail elderly syndrome (00231) to 2,775 (38.6%) for sedentary lifestyle (00168). The first column of data in Table 3 shows the sizes of these subsamples for each of the 15 diagnostic labels obtained. Table 3 also shows the results of the NANDA‐I estimation of the association between level of vulnerability and diagnosis by comparing the relative frequencies of assignment of each diagnostic label among level of vulnerability and the estimation of the absolute and age‐ and sex‐adjusted risks of receiving a diagnosis as a function of belonging to the most vulnerable group.

**Table 3:** Association between NANDA‐I diagnoses taken from the CDC‐Canarias database and social class according to the ICE index

Diagnostics	Total*n* (%)	Poor class status*n* (%)	Wealthy class status*n* (%)	*P* ^1^	Crude risk (CI 95%)^2^	Sex‐ and age‐adjusted risk (CI 95%)^3^
00276 Ineffective health self‐management	1157 (16.0)	951 (82.2)	206 (17.8)	<0.001	2.42 (2.05–2.86)	1.41 (1.18–1.70)
00292 Ineffective health maintenance behaviors	1579 (21.9)	1141 (72.3)	438 (27.7)	0.002	1.18 (1.03–1.36)	1.22 (1.06–1.42)
00168 Sedentary lifestyle	2775 (38.6)	2099 (75.6)	676 (24.4)	<0.001	2.08 (1.81–2.38)	1.94 (1.68–2.23)
00188 Risk‐prone health behavior	1355 (18.8)	930 (68.6)	425 (31.4)	0.003	0.90 (0.79–1.04)	1.06 (0.91–1.23)
00215 Deficient community health	773 (10.7)	642 (83.1)	131 (16.9)	<0.001	2.39 (1.96–2.92)	1.26 (1.02–1.57)
00231 Risk for frail elderly syndrome^4^	132 (1.8)	124 (93.9)	8 (6.1)	<0.001	6.92 (3.38–14.20)	0.65 (0.16–2.63)
00179 Risk for unstable blood glucose level	189 (2.6)	163 (86.2)	26 (13.8)	0.002	2.78 (1.83–4.22)	1.44 (0.93–2.24)
00232 Obesity	1210 (16.8)	1028 (85.0)	182 (15.0)	<0.001	3.15 (2.64–3.74)	2.46 (2.05–2.95)
00233 Overweight	1606 (22.3)	1155 (71.9)	451 (28.1)	0.002	1.15 (1.00–1.32)	0.98 (0.85–1.14)
00234 Risk for overweight	525 (7.2)	311 (59.2)	214 (40.1)	0.013	0.57 (0.48–0.69)	0.88 (0.72–1.07)
00011 Constipation	1184 (16.4)	824 (69.6)	360 (30.4)	0.002	0.97 (0.83–1.12)	0.95 (0.81–1.12)
00015 Risk for constipation	236 (3.2)	202 (85.6)	34 (14.4)	<0.001	2.65 (1.83–3.83)	1.38 (0.94–2.04)
00299 Risk for decreased activity intolerance	855 (11.9)	599 (70.0)	256 (30.0)	<0.001	1.00 (0.85–1.18)	1.00 (0.84–1.19)
00311 Risk for impaired cardiovascular function^3^	820 (11.4)	694 (84.6)	126 (15.4)	<0.001	2.76 (2.25–3.38)	1.60 (1.29–1.99)
00303 Risk for adult falls	682 (9.4)	509 (74.6)	173 (25.4)	0.004	1.31 (1.09–1.57)	1.16 (0.95–1.41)

^1^ Estimated with Pearson chi‐squared test.

^2^ Estimated with univariate binary logistic regression model.

^3^ Estimated with multivariate binary logistic regression model.

^4^ Over 65 years old.

## DISCUSSION

The results of the present study confirm the socio‐epidemiological hypothesis that nursing care needs do not follow a random distribution among the population, but instead are preeminent among the most vulnerable within the population. Health inequalities arise from the social conditions in which people are born, develop, live, work, and age (WHO, 2008). These factors socially condition a person's health. This research also helps nurses to determine nursing care needs, identified with NANDA‐I terminology.

### Domain 1. Health promotion

The most prevalent diagnosis in the sample here is sedentary lifestyle (00188), from NANDA‐I Domain 1. Six out of 10 cases presented it, mainly women and middle‐aged people. Physical inactivity is the fourth risk factor in mortality. Adults (23%) and adolescents (81%) do not get enough physical exercise (WHO, 2018). In Spain, one third of the population do not engage in physical activity, and almost half do so occasionally (Ministry of Education, Culture and Sports, 2016). These figures are similar in the Canary Islands and in the sample studied here. There is a strong correlation of this diagnosis with the vulnerable position, coinciding with other studies in which young people with a higher level of education do more physical activity (Maestre‐Miquel et al., 2015).

Ineffective health maintenance behaviors (00292) have been identified in one out of every three participants, with a higher prevalence in men and a strong correlation with the most vulnerable position. Lemstra, Blackburn, Crawley, and Fung (2012) reported that better adherence to treatment would save more lives than new drugs. It is essential for nurses to identify factors that influence adherence to self‐care and to intervene when problems in health maintenance are detected and thus avoid serious complications requiring hospitalization (Carneiro et al., 2018).

Special attention is required for populations identified as socioeconomically vulnerable, whose possibilities of buying medication and healthy food are low. A systematic review (Nieuwlaat et al., 2014) concluded that current methods to improve adherence to chronic health problems are complex and not useful. Not considering underlying socioeconomic factors when prescribing medication or lifestyle changes may be a significant cause of failure.

Other diagnoses in Domain 1 present in our sample of vulnerable people were risk‐prone health behavior (00188) and ineffective health self‐management (00276). The former was more prevalent in men and young people aged 18–39, and this is consistent with the research of Korn and Bonny‐Noach (2018). The latter was more frequently identified in men and people aged > 65. Carneiro et al. (2018) suggest that nonadherence in higher in men may be attributed to women being more attentive to their health and health needs because as primary caregivers, they need to be healthy to care for others. They indicate that low educational and socioeconomic status contributes to poor health management, increasing exacerbations. Little evidence supports the effectiveness of interventions for patients and people with high social vulnerability (Van Hecke et al., 2017). As clinicians close to the patient, nurses are uniquely positioned to address educational and motivational strategies for self‐care, identify barriers, and promote interventions in disadvantaged populations.

Deficient community health (00215) is a critical population surveillance diagnosis, especially in our sample of vulnerable populations, women, and older groups. Its presence indicates an absence of well‐being and increased risk of new problems.

This calls for the activation of community prevention mechanisms by correcting unhealthy practices and maintaining safe environments. The diagnosis of specific population needs allows for the programming of public health interventions in associations, schools, institutes, and community meeting places. Adolescent health should be promoted, and diseases should be prevented, including chronic illness and sexually transmitted diseases. Additional health concerns that should be addressed in adolescents and in consideration of inequities include reproductive health, vaccinations, smoking, alcoholism, and substance misuse, as well as mental health problems (Peckham et al., 2017). The identification of the nursing diagnosis has been described as having the potential to facilitate the involvement and activation of community members for their own health, participating in the design of services they need (Coulter, 2010; Hibbard & Greene, 2013).

The risk for frail elderly syndrome (00231) is also more frequent in vulnerable populations, and it is essential to consider their psychosocial needs, especially from the perspective of PHC, because social isolation and hopelessness usually exist as part of this syndrome (Ribeiro et al., 2019). This diagnosis gives nurses a comprehensive vision of the older adult and a new approach to biopsychosocial needs.

### Domain 2. Nutrition

Three out of four participants had some type of diagnosis of obesity (00232), overweight (00233), or risk for overweight (00234) in nutrition Domain 2 of NANDA‐I. The first two are concentrated in the mature age group while the risk for overweight (00234) is concentrated among young people. Obesity is a major global issue with health consequences that must be addressed early (GBD 2015 Obesity Collaborators, 2017). Obesity is determined by multiple factors: political–historical, cultural, residence, socioeconomic level, and genetic predisposition.

It is often identified between generations, and the disadvantaged socioeconomic position is a determining factor in suffering from it throughout life (Panetta & López‐Valcárcel, 2016). The condition often begins in childhood and adolescence and has environmental and behavioral components (Duarte‐Salles et al., 2011). Educational level has been identified as a factor with the most significant impact (Rodríguez‐Caro et al., 2016). The risk for unstable blood glucose levels (00179) was also more frequent in the most vulnerable and in the elderly, coinciding with other studies that mention the relevance of considering new risk factors (Teixeira et al., 2017).

### Domain 3. Elimination and exchange

Almost one in three participants present constipation (00011) or risk for constipation (00015), diagnoses in Domain 3, elimination and exchange, which is consistent with the prevalence in the general population. This finding is consistent with other work, indicating 30% of the population experience this at some point during life with a predominance in women (De Giorgio et al., 2015). This condition is one of the most frequent gastrointestinal disorders, also in older people. The present study identified the risk for constipation (00015) associated with older age. On the other hand, constipation (00011) was more frequent in younger age groups, which may be related to how the data of the “CDC‐Canarias” study were collected, directly declared by the participants, being perceived, and referred to a greater extent by young people, while the elderly may have associated this symptomatology with problems of age, without giving it enough importance. Patients' perspective on constipation should be integrated into the actions of health professionals (Tvistholm et al., 2017). It is not clinically relevant until it generates risks and affects the quality of life (Sun et al., 2011), and has a considerable impact when it becomes chronic, negatively affecting healthcare costs. Providing information on fiber‐rich diets and bowel training are effective nursing interventions (Bardsley, 2017).

### Domain 4. Activity/rest

Concerning diagnoses in Domain 4, risk of activity intolerance (00094) was more common among women and the most vulnerable ones, but similar according to age. The risk for decreased activity intolerance (00299) is strongly related to social vulnerability, and its prevalence increases with age for both men and women. Inequalities in cardiovascular health have been associated with indicators of education, income, and occupation (Havranek et al., 2015). Unemployment, multiple job losses, subjective perception of social status, and residential neighborhood deprivation have been associated with cardiovascular risk and increased mortality (Dupre et al., 2012; Ramsay et al., 2015). The sample here confirms the disproportionate prevalence of diagnosis among the most vulnerable.

### Domain 11. Safety/protection

The prevalence of risk for adult falls (00303), Domain 11 safety‐protection, increases with age and is associated with vulnerable populations. Falls, especially in the elderly, are a priority public health problem, with physical, psychological, social, and economic consequences. Some risk factors are modifiable, at both the individual and community levels (Moncada & Mire, 2017). Sousa et al. (2017) pointed out the need to review this nursing diagnosis, including risk factors such as socioeconomic ones, a relationship verified in the study here. The most frequent repercussions of falls are reduced mobility, loss of autonomy and quality of life, increased morbidity and mortality, and a higher probability of institutionalization. Epidemiological knowledge of the prevalent repercussions of falls in the Spanish population (Pellicer García et al., 2015), and the information reported in the research here may contribute to preventing such repercussions.

To the best of the authors' knowledge, cross‐mapping is applied here for the first time to systematically compare individual and compound terms from the terminology used in a database of a medical–epidemiological nature with their semantic equivalents in the NANDA‐I classification terminology. Cross‐mapping has been previously applied as a method to link records made by nurses and standardized languages (D'Agostino et al., 2020; Frauenfelder et al., 2016; Goossen, 2006) and to develop equivalencies between classifications (Kim et al., 2014). Likewise, by applying this method, correspondence has been found between different nursing languages and clinical fields (Juvé Udina et al., 2012).

## LIMITATIONS

The present study has some limitations. The first limitation is that the information available in the “CDC‐Canarias” database is designed for a study restricted to medical epidemiology, and therefore it only identified nursing diagnoses related to physiological–functional domains of NANDA‐I. Another limitation is that the component aspects of the diagnoses contained in the “CDC‐Canarias” database were obtained through self‐completed questionnaires, which did not include nursing assessment, clinical decision‐making, or judgment. The findings were dependent upon the veracity of the content declared by the participants. As a third limitation, the study was restricted to the availability of samples from the “CDC‐Canarias” database, made up of subjects who fulfilled the condition of having a double label, that of the NANDA‐I diagnosis and that of extreme ICE index of vulnerability. This limited the sample sizes available for multivariate analysis that may have notably reduced the conditions of optimal applicability for these models. This introduces reasonable doubt about whether the associations between vulnerability and NANDA‐I diagnosis are due to their nonexistence or a loss of the power of the study in these cases. However, the study also has strengths. The first is that the risks found in the identification of associations between vulnerability and NANDA‐I diagnoses are not affected by loss of power beyond a statistical significance attributable to large sample sizes. The majority of the cases here are relevant and seem to confirm the connection. The second strength of the study is conceptual: to the best of the authors' knowledge, the study is the first attempt to assess the association between social vulnerability and nursing care needs using data of a medical nature.

## IMPLICATIONS FOR NURSING KNOWLEDGE AND/OR LANGUAGE DEVELOPMENT

This research provides knowledge about the relationship between vulnerable populations and nursing care needs, expressed in the diagnostic terminology of the NANDA‐I classification, based on sociodemographic, clinical, and lifestyle data from a representative cohort of the Canary population, Spain. From an epidemiological perspective, the study provides information on care needs in the domains of health promotion, nutrition, elimination–exchange, activity–rest, and safety/protection, thereby facilitating a posteriori adequate planning of interventions for the identified population profiles. More work is needed to cross map actual and potential health‐related nursing problems considering individual and community vulnerability. Identifying the distribution of nursing diagnoses may help nurses to measure/address the needs of the population and as a result allocate adequate resources to optimize the health of the population.

## CONCLUSIONS

Bearing in mind the above limitations, all NANDA‐I diagnoses in the study were found to be most often associated with this vulnerable populations. However, to confirm these findings and expand their scope following the strategy of exploiting available data, new studies are required to identify NANDA‐I nursing diagnoses by mapping sociodemographic and clinical data contained in cohort study databases on representative samples of social vulnerability and geographical location of populations, which would allow a further step to be taken in identifying the most prevalent nursing care needs and their niches in order to plan community nursing interventions, implement them, and assess their impact.

### Knowledge translation

- This article presents a mapping of information from a population cohort to identify NANDA‐I nursing diagnoses based on terminological equivalencies, by consensus with experts and by constructing qualitative rules for their assignment.
- Fifteen diagnostic rules are drawn up, eight diagnoses focus on problems and seven on risk, describing the prevalence among the Canary population.
- The study describes the differences identified in the frequency of problems concerning social vulnerability, sex, and age of the participants.

## CONFLICT OF INTERESTS

No conflict of interest were declared.

## ETHICS STATEMENT

The Research Ethics Committee approved the study, and the participation of the experts in the expert panel took place after signing the Informed Consent Term forms.

## AUTHOR CONTRIBUTION

Fernández‐Gutiérrez, Brito‐Brito and Darias‐Curvo contributed substantially to the conception and design of the study, Cabrera‐de‐León, Aguirre‐Jaime, Brito‐Brito, Martínez‐Alberto and Fernández‐Gutiérrez contributed to the data acquisition, analysis and interpretation. All the authors have drafted or provided critical revision of the manuscript. They also agreed to be accountable for all aspects of the work in ensuring that questions to the accuracy or integrity of any part of the work are appropriately investigated and resolved.
